# Changing trends in the disease burden of primary liver cancer caused by specific etiologies in China

**DOI:** 10.1002/cam4.2477

**Published:** 2019-08-06

**Authors:** Zhenqiu Liu, Xianhua Mao, Yanfeng Jiang, Ning Cai, Li Jin, Tiejun Zhang, Xingdong Chen

**Affiliations:** ^1^ State Key Laboratory of Genetic Engineering and Collaborative Innovation Center for Genetics and Development, School of Life Sciences Fudan University Shanghai China; ^2^ Fudan University Taizhou Institute of Health Sciences Taizhou China; ^3^ Key Laboratory of Public Health Safety, Department of Epidemiology, Ministry of Education, School of Public Health Fudan University Shanghai China; ^4^ Human Phenome Institute Fudan University Shanghai China

**Keywords:** alcohol use, China, HBV, HCV, incidence, liver cancer, mortality, NASH

## Abstract

**Background:**

Liver cancer is a commonly diagnosed malignancy in China. The etiologies of liver cancer are widely known, although studies on temporal trends in liver cancer caused by specific etiologies are rare.

**Methods:**

Data on the incidence and mortality of liver cancer were retrieved from the Global Burden of Diseases Study 2017. The estimated annual percentage change (EAPC) was used to quantify temporal trends in the age‐standardized incidence rate (ASIR) and the age‐standardized mortality rate (ASMR) of liver cancer from 1990 to 2017.

**Results:**

Nationwide, the number of incident cases of liver cancer increased from 258 000 in 1990 to 515 900 in 2017. The ASIR decreased from 27.16 per 100 000 to 26.04 per 100 000 during this period, with an EAPC of −0.64 (95% confidence interval [CI] −0.84, −0.44). The number of deaths increased from 245 300 in 1990 to 418 200 in 2017, and the ASMR decreased from 26.72 to 21.30 (EAPC = −1.16, 95% CI −1.35, −0.97). The most pronounced decreases in the ASIR and ASMR were observed in liver cancer due to hepatitis B and in people aged 15‐49 years.

**Conclusions:**

Since the extensive efforts for prevention of hepatitis B virus infection, the incidence of liver cancer due to hepatitis B has significantly decreased. However, liver cancer due to hepatitis C, NASH, and other causes remains a major public health concern. Additional preventive strategies tailored to liver cancer are needed to further reduce its disease burden in China.

## Graphical abstract

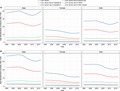

## 1 INTRODUCTION

Liver cancer is predicted to have been the sixth most commonly diagnosed cancer and the fourth leading cause of cancer‐related death worldwide in 2018, with approximately 841 000 new cases and 782 000 deaths occurring annually.^1^ Worldwide, more than half of newly diagnosed cancer cases and deaths have occurred in China.^2^, ^3^, ^4^, ^5^ According to the 2015 cancer statistics, 466 100 and 422 100 new liver cancer cases and deaths, respectively, were estimated to have occurred in China.^3^

Etiologies underlying liver cancer have been extensively investigated in the past decade.^6^, ^7^, ^8^, ^9^ Hepatitis virus infections, including hepatitis B virus (HBV) and hepatitis C virus (HCV), have been identified as major contributors to liver cancer.^8^, ^10^ Accordingly, the Chinese government has initiated measures to combat the hepatitis epidemic. For example, free‐of‐charge HBV vaccinations have been provided for all newborns since 2005, and a HBV vaccination “catch‐up” program commenced in 2009 for children aged 8‐15 years.^11^ These efforts have markedly reduced the HBV surface antigen (HBsAg) carrier rate in the general population.^12^, ^13^ To prevent HCV transmission, China enacted a blood donation law in 1998 and implemented a volunteer blood donation system, which further improved the quality of donated blood.^14^ However, obtaining accurate estimations of the prevalence of HBV and HCV is challenging due to limited access to the real‐world infection reservoir.^15^ According to our previous study, which was based on China's national web‐based surveillance system, the reported incidence of HBV remained stable from 2004 to 2014, while the incidence of HCV rapidly increased during this period.^16^ These findings not only reflected the gradual implementation of this surveillance system and the rise in healthcare availability but also warned that HBV and HCV remained major public health concerns in China. Apart from HBV and HCV, several other factors, such as alcohol consumption,^17^ nonalcoholic fatty liver disease (NAFLD), and nonalcoholic steatohepatitis (NASH),^18^, ^19^ have been identified as major risk factors for liver cancer. More importantly, certain factors that also contribute to the development of liver cancer might be obscured or ignored. Consequently, determining the etiological pattern of liver cancer is critical for its precise prevention.

The Global Burden of Diseases (GBD) study described the global landscape of the burden of primary liver cancer and provided an unprecedented opportunity for us to understand the cancer burden in China.^20^ In this study, we analyzed temporal trends in liver cancer caused by several distinct etiologies by age and sex from 1990 to 2017. Our results were intended to facilitate evidence‐based planning and evaluation of the effectiveness of current prevention and treatment strategies, as well as the assessment of future disease burden and subsequent allocation of limited healthcare resources.

## 2 MATERIALS AND METHODS

### 2.1 Study data

The following data were retrieved from the Global Health Data Exchange query tool (http://ghdx.healthdata.org/gbd-results-tool)^21^: annual incidence, age‐standardized incidence rate (ASIR), number of deaths and the age‐standardized mortality rate (ASMR) of liver cancer in China from 1990 to 2017 by sex, age, and etiology (hepatitis B, hepatitis C, alcohol consumption, NASH, and other causes). Since the dearth of cancer case in people aged <15 years, especially for liver cancer due to etiology other than hepatitis B, we only retrieved the data for the remaining population (ie, 15‐49, 50‐69, and ≥70 years). The general methods for the 2017 GBD study have been reported in detail in previous studies.^20^, ^22^ The liver cancer incidence in the GBD dataset was estimated by the following ways: (a) the mortality to incidence ratio (MIR) for liver cancer was estimated by using any data sources that report both, incidence and mortality, and predict the MIR for all locations using the Healthcare Access and Quality Index; (b) any incidence data were sought from individual cancer registries or aggregated databases of cancer registries, for example, Cancer Incidence in Five Continents, Surveillance, Epidemiology, and End Results, and NORDCAN. All ICD9 and ICD10 codes pertaining to PLC (155‐155.963 and C22.0‐9, respectively) were included in these estimates; (c) the cancer registry incidence data were multiplied with the MIR to estimate liver cancer mortality; (d) the liver cancer mortality estimates were added to liver cancer mortality data from vital registration system data (death certificates); (e) these combined data sources are the input for a “CODEm” model (Cause of Death ensemble model), where the covariates were used to predict liver cancer mortality for all locations, ages, sexes, and years; (f) the liver cancer mortality estimates were adjusted to the separately estimated all‐cause mortality; (g) these adjusted liver cancer deaths were divided by the MIR estimates to determine liver cancer incidence. Data were excluded if they were not representative of the respective population (eg, hospital‐based registries), if they did not cover all malignant neoplasms as defined by the ICD9 (140‐280) or ICD10 codes (C00‐C96), if they did not include data for both sexes and all age groups, if the data were limited to a period before 1980, or if the source did not provide details on the population included in the dataset. Preference was given to registries with national coverage over those with only local coverage, with the exception of registries from countries for which the GBD study provided subnational estimates. A detailed description of the sources of the mortality data and the processing steps followed to develop the cause‐of‐death database can be found in the appendix of the systematic review by Wang et al.^23^ Briefly, a majority of the cause‐of‐death data are vital registration data obtained from either the WHO mortality database or country‐specific mortality databases operated by official offices provided by trusted country collaborators. The two primary sources of data for China were surveillance data from the China Disease Surveillance Points (DSP) system and vital registration data collected by the Chinese Center for Disease Control and Prevention (CDC). In the China DSP data, deaths were reported across 145 DSP that were used from 1991 to 2003 and 161 points that were used from 2004 to 2007. While the China DSP data derived from ICD10 codes are considered surveillance data, they provide national coverage and details related to the cause of death. Thus, the China DSP data are processed and treated in a similar way as the data from the China CDC vital registration from 2008 to 2012. From 2008 to 2012, all of the deaths and cause‐of‐death information from the DSP system and other systems throughout China were collected and reported via the Mortality Registration and Reporting System, an online reporting system of the Chinese CDC. The data were processed using “CODEm” model (Cause of Death ensemble model). CODEm is a framework for modeling most cause‐specific death rates in the GBD using four core principles: (a) Identify and use all the available data in the modeling process. Though data may vary in quality, they all contain some signal of the true epidemiological process. (b) Develop a diverse set of plausible models to use for estimation. That is, build a number of models capturing well‐documented associations to make estimates. (c) Assess the predictive validity of each plausible individual model and of an ensemble of models created from the pool of plausible models. (d) Choose the models and ensemble model with the best performance in the out‐of‐sample predictive validity tests.

A systematic search of the relevant literature was performed using PubMed to ascertain the proportion of liver cancer cases due to each of the five etiologies included in the GBD. Studies were included if the study population was representative of liver cancer patients in the respective location. The proportions of liver cancer due to the four specific etiologies were calculated, and the remaining etiologies were combined in an “other causes” group. The proportion data obtained from the systematic literature review were used as input for the four separate DisMod‐MR 2.1 models to determine the proportion of liver cancer due to the four etiologies for all locations, both sexes, and all age groups.^20^

### 2.2 Statistical analysis

The estimated annual percentage change (EAPC) was used to quantify the trends in the ASIR and ASMR of liver disease and was stratified by sex, etiology, and age from 1990 to 2017. The EAPC is a summative and widely used measure of the ASIR/ASMR trend over a specified interval. A regression line was fitted to the natural logarithm of the rates, that is, *y = α + βx + ɛ*, where *y = *ln(ASIR[ASMR]), and *x* = calendar year. The EAPC was calculated as 100 × (*exp*(*β*) − 1), and its 95% confidence interval (CI) was also obtained from the linear regression model.^24^ All statistical analyses were performed using the R program (Version 3.5.1, R core team). A *P‐*value less than .05 was considered statistically significant.

## 3 RESULTS

### 3.1 Liver cancer in China

**Figure 1:**
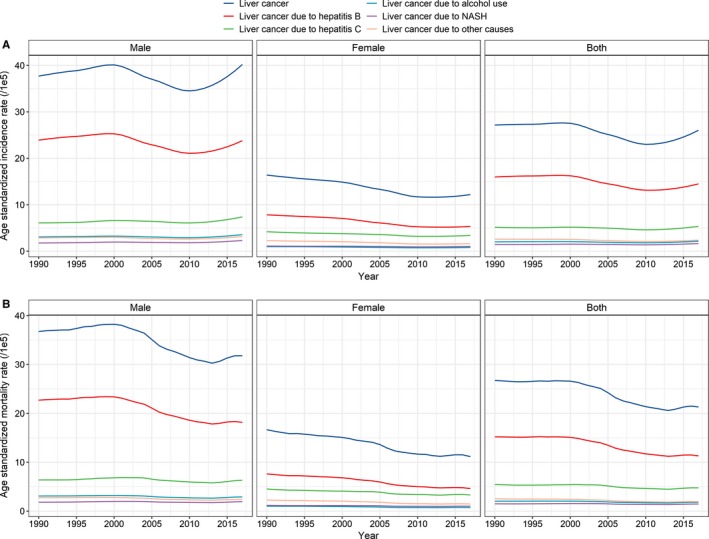
Temporal trends in the A, age‐standardized incidence rate and B, age‐standardized mortality rate by sex and etiology in China from 1990 to 2017

**Figure 2:**
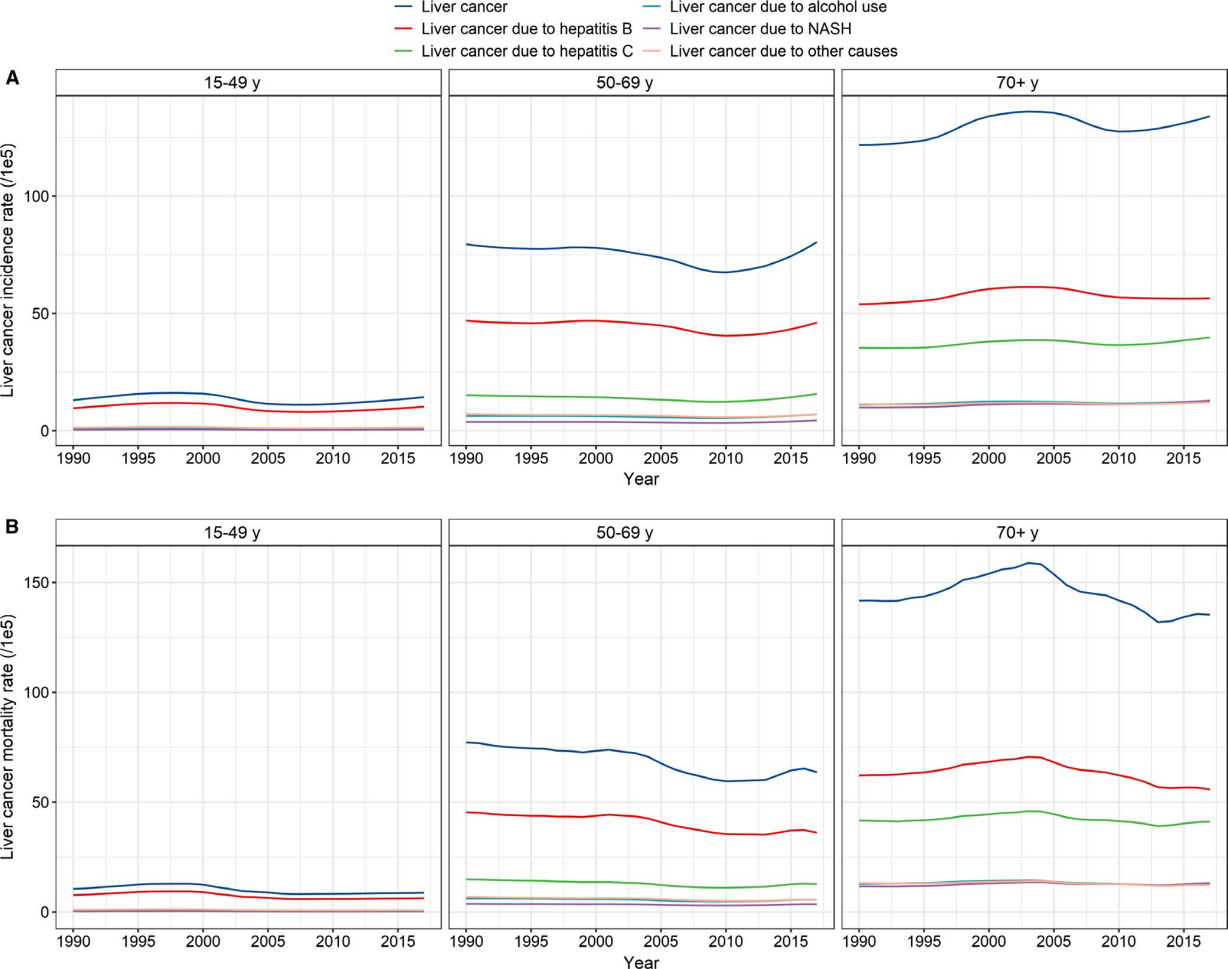
Temporal trends in the A, age‐standardized incidence rate and B, age‐standardized mortality rate by age and etiology in China from 1990 to 2017

The number of incident cases of liver cancer increased nationwide from 258.0 thousand (95% uncertainty interval [UI] 240.2, 272.8) in 1990 to 515.9 thousand (95% UI 486.2, 546.2) in 2017. The ASIR decreased from 27.16 (95% UI 25.31, 28.69) per 100 000 to 26.04 (95% UI 24.57, 27.54) per 100 000 during this period, with an EAPC of −0.64 (95% CI −0.84, −0.44) (Table 1, Figure 1A). Cancer rates among males increased more than twofold between 1990 and 2017 (from 182 700 to 395 200), whereas the ASIR showed a mild decrease (from 37.70 per 100 000 to 40.19 per 100 000, EAPC = −0.23, 95% CI −0.06, −0.49) during the same period. A more pronounced decrease among females was observed in the ASIR (from 16.39 per 100 000 to 12.19 per 100 000, EAPC = −1.47, 95% CI −1.30, −1.63), although the number of cancer cases increased from 75.3 thousand to 120.7 thousand (Table 1, Figure 1A). The incidence showed an upward trend among people aged ≥70 years (EAPC = 0.24, 95% CI 0.09, 0.40) (Table 1, Figure 2A).

**Table 1:** The incident cases and age‐standardized incidence of primary liver cancer in 1990 and 2017 and its temporal trends from 1990 to 2017

Characteristics	1990		2017		1990‐2017
	Incident cases No. ×10^3^ (95% UI)	ASIR per 100 000 No. (95% UI)	Incident cases No. ×10^3^ (95% UI)	ASIR per 100 000 No. (95% UI)	EAPC in ASIR No. (95% CI)
Overall	258.0 (240.2, 272.8)	27.16 (25.31, 28.69)	515.9 (486.2, 546.2)	26.04 (24.57, 27.54)	−0.64 (−0.84, −0.44)
Sex					
Male	182.7 (164.5, 196.7)	37.70 (34.09, 40.59)	395.2 (366.1, 426.8)	40.19 (37.30, 43.37)	−0.23 (−0.06, −0.49)
Female	75.3 (65.7, 83.6)	16.39 (14.32, 18.25)	120.7 (111.3, 132.3)	12.19 (11.25, 13.36)	−1.47 (−1.30, −1.63)
Age^a^					
15‐49 years	87.3 (78.8, 93.9)	13.06 (11.80, 14.05)	106.8 (98.3, 116.1)	14.35 (13.22, 15.61)	−0.85 (−1.39, −0.30)
50‐69 years	122.4 (114.5, 129.0)	79.46 (74.34, 83.74)	276.6 (258.6, 295.2)	80.39 (75.16, 85.80)	−0.40 (−0.61, −0.18)
70+ years	47.1 (44.1, 50.3)	121.76 (114.08,130.17)	132.0 (124.4, 140.0)	134.06 (126.33, 142.14)	0.24 (0.09, 0.40)
Etiology					
Hepatitis B	158.4 (146.6, 169.1)	15.98 (14.79, 17.05)	290.9 (270.9, 312.2)	14.49 (13.53, 15.54)	−0.88 (−1.09, −0.67)
Hepatitis C	43.8 (40.1, 47.3)	5.14 (4.73, 5.52)	103.1 (95.1, 111.8)	5.34 (4.94, 5.77)	−0.22 (−0.39, −0.04)
Alcohol use	18.6 (15.1, 23.0)	2.02 (1.66, 2.47)	43.3 (35.0, 53.9)	2.16 (1.76, 2.66)	−0.27 (−0.50, −0.05)
NASH	12.5 (11.0, 14.0)	1.44 (1.27, 1.61)	31.7 (27.9, 35.4)	1.66 (1.46, 1.84)	0.08 (−0.12, 0.29)
Other causes	24.8 (21.5, 27.7)	2.59 (2.28, 2.88)	46.8 (41.7, 52.4)	2.39 (2.14, 2.66)	−0.81 (−1.03, −0.59)

Abbreviations: ASIR: age‐standardized incidence rate; CI: confidence interval; EAPC: estimated annual percentage change; NASH: nonalcoholic steatohepatitis; UI: uncertainty interval.

a Crude mortality rate in each age group.

Table 2 presents the numbers of liver cancer‐related deaths and the ASMRs in 1990 and 2017 and the temporal trends in the ASMR from 1990 to 2017. Overall, the number of deaths increased from 245.3 thousand (95% UI 228.3, 258.9) in 1990 to 418.2 thousand (95% UI 396.4, 441.2) in 2017, and the ASMR decreased from 26.72 (95% UI 24.93, 28.19) to 21.30 (95% UI 20.21, 22.44) (Table 2, Figure 1B). Significant decreases in the ASMR were found in both males and females and in all age groups (Table 2, Figure 2B).

**Table 2:** The number of deaths and age‐standardized mortality rates of primary liver cancer in 1990 and 2017 and the temporal trends from 1990 to 2017

Characteristics	1990		2017		1990‐2017
	Deaths No. ×10^3^ (95% UI)	ASMR per 100 000 No. (95% UI)	Deaths No. ×10^3^ (95% UI)	ASMR per 100 000 No. (95% UI)	EAPC in ASMR No. (95% CI)
Overall	245.3 (228.3, 258.9)	26.72 (24.93, 28.19)	418.2 (396.4, 441.2)	21.30 (20.21, 22.44)	−1.16 (−1.35, −0.97)
Sex					
Male	170.9 (154.5, 184.1)	36.74 (33.25, 39.60)	308.3 (288.5, 331.0)	31.80 (29.80, 34.12)	−0.90 (−1.11, −0.70)
Female	74.4 (65.0, 82.9)	16.65 (14.59, 18.63)	109.9 (102.2, 119.9)	11.15 (10.38, 12.16)	−1.67 (−1.83, −1.52)
Age^a^					
15‐49 years	70.6 (63.8, 75.7)	10.56 (9.55, 11.34)	65.5 (61.1, 70.1)	8.80 (8.21, 9.42)	−1.69 (−2.20, −1.19)
50‐69 years	118.9 (111.1, 125.2)	77.19 (71.10, 81.27)	219.1 (206.7, 232.6)	63.69 (60.08, 67.61)	−0.99 (−1.20, −0.78)
70+ years	54.8 (51.4, 58.6)	141.72 (132.95, 151.65)	133.3 (127.1, 140.7)	135.34 (129.08, 142.86)	−0.28 (−0.52, −0.03)
Etiology					
Hepatitis B	146.5 (135.4, 156.1)	15.20 (14.10, 16.18)	226.4 (211.9, 241.5)	11.31 (10.59, 12.03)	−1.56 (−2.94, −0.16)
Hepatitis C	44.7 (41.0, 48.1)	5.44 (5.03, 5.86)	90.3 (83.6, 97.8)	4.77 (4.43, 5.15)	−0.79 (−1.37, −0.21)
Alcohol use	18.3 (15.0, 22.5)	1.53 (1.25, 1.88)	35.8 (29.2, 44.4)	1.81 (1.49, 2.23)	−0.98 (−2.35, 0.41)
NASH	12.5 (11.0, 14.1)	1.50 (1.33, 1.70)	27.6 (24.4, 30.8)	1.47 (1.30, 1.63)	−0.44 (−1.06, 0.18)
Other causes	23.3 (20.4, 25.9)	2.54 (2.23, 2.81)	37.9 (34.1, 42.3)	1.95 (1.76, 2.16)	−1.35 (−1.76, −0.95)

Abbreviations: ASIR: age‐standardized incidence rate; CI: confidence interval; EAPC: estimated annual percentage change; NASH: nonalcoholic steatohepatitis; UI: uncertainty interval.

a Crude mortality rate in each age group.

### 3.2 Liver cancer due to hepatitis B

**Figure 3:**
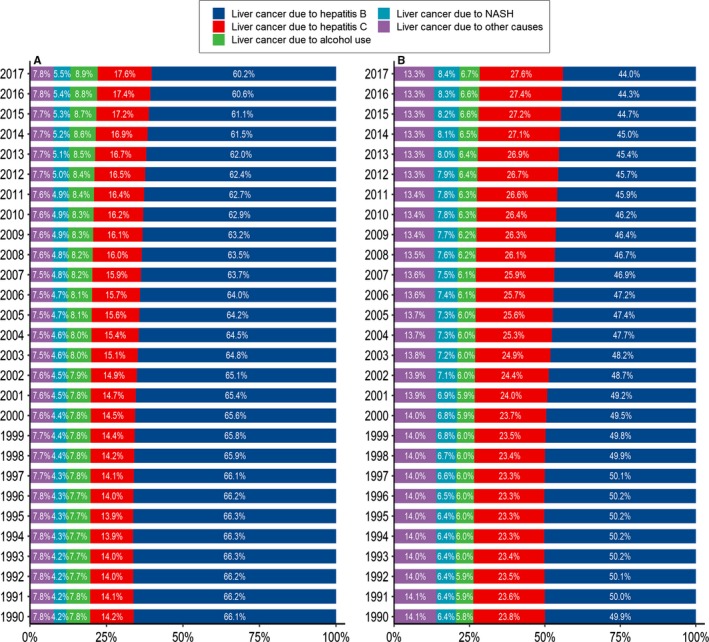
Proportions of liver cancer due to specific etiologies in China from 1990 to 2017. (A: in males and B: in females)

In 2017, 290.9 thousand newly diagnosed cases and 226.4 thousand deaths were ascribed to liver cancer due to hepatitis B (Tables 1 and 2). The ASIR of liver cancer due to hepatitis B decreased from 15.98 per 100 000 in 1990 to 14.49 per 100 000 in 2017, with an EAPC of −0.88 (95% CI −1.09, −0.67) (Figure 1A), and a corresponding decrease from 15.20 per 100 000 to 11.31 per 100 000 was found in the ASMR during the study period, with an EAPC of −1.56 (95% CI −2.94, −0.16) (Figure 1B). The proportion of liver cancer due to hepatitis B cases among males decreased from 66.1% in 1990 to 60.2% in 2017 (Figure 3). However, only a mild reduction was found in the ASIR from 1990 to 2017 (EAPC = −0.55, 95% CI −0.76, −0.33) (Figure 1A, Table 3). Notably, a significant upward trend was observed in the ASIR after 2010 (EAPC = 1.77, 95% CI 1.30, 2.24). An upward trend in the ASMR was observed between 1990 and 2000, but thereafter, the ASMR showed a marked decrease, with an EAPC of −0.95 (95% CI −1.37, −0.52) (Figure 1B, Table 4). The proportion of liver cancer due to hepatitis B cases among females decreased from 49.9% to 44.0% (Figure 3, Table 3), with a marked decrease in the ASIR during the study period (EAPC = −1.85, 95% CI −2.02, −1.67). However, the downward trend leveled off after 2010 (EAPC = 0.15, 95% CI −0.20, 0.49) (Figure 1A). Unlike the ASMR of the males, the ASMR of the females showed a steady decrease from 1990 to 2017 (EAPC = −2.07, 95% CI −2.24, −1.89) (Figure 1B, Table 4). A significant decrease was observed in the incidence of liver cancer due to hepatitis B in people aged 15‐49 and 50‐69 years but not in people aged ≥70 years (EAPC = −0.98 [95% CI −1.53, −0.43]; −0.43 [95% CI −0.61, −0.25]; 0.11 [95% CI −0.09, 0.31], respectively) (Figure 2A, Table 3). A significant reduction in mortality was found in all three age groups (Figure 2B, Table 4).

**Table 3:** The incident cases and age‐standardized incidence of primary liver cancer by etiology in 1990 and 2017 and the temporal trends from 1990 to 2017

Characteristics	1990		2017		1990‐2017
	Cases No. ×10^3^ (95% UI)	ASIR per 100 000 No. (95% UI)	Cases No. ×10^3^ (95% UI)	ASIR per 100 000 No. (95% UI)	EAPC in ASIR No. (95% CI)
*Liver cancer due to hepatitis B*					
Sex					
Male	120.7 (108.7, 130.2)	23.91 (21.51, 25.74)	237.9 (219.6, 257.9)	23.79 (21.99, 25.80)	−0.55 (−0.76, −0.33)
Female	37.6 (32.5, 42.0)	7.83 (6.78, 8.75)	53.1 (48.3, 58.6)	5.32 (4.84, 5.88)	−1.85 (−2.02, −1.67)
Age^a^					
15‐49 years	64.3 (57.9, 69.5)	9.62 (8.67, 10.40)	76.3 (69.6, 83.5)	10.25 (9.35, 11.22)	−0.98 (−1.53, −0.43)
50‐69 years	72.3 (66.2, 77.5)	46.91 (42.99, 50.28)	158.7 (146.4, 172.5)	46.12 (42.54, 50.15)	−0.43 (−0.61, −0.25)
70+ years	20.8 (19.0, 22.7)	53.92 (49.15, 58.83)	55.6 (51.3, 60.3)	56.48 (52.13, 61.26)	0.11 (−0.09, 0.31)
*Liver cancer due to hepatitis C*					
Sex					
Male	25.9 (22.9, 28.7)	6.09 (5.42, 6.72)	69.7 (63.0, 77.0)	7.38 (6.71, 8.12)	0.30 (0.10, 0.49)
Female	17.9 (15.5, 20.2)	4.19 (3.65, 4.75)	33.3 (30.3, 37.0)	3.40 (3.10, 3.77)	−1.02 (−1.15, −0.89)
Age^a^					
15‐49 years	6.7 (5.8, 7.5)	1.00 (0.88, 1.13)	9.7 (8.4, 11.0)	1.30 (1.12, 1.47)	−0.09 (−0.62, 0.45)
50‐69 years	23.4 (21.0, 25.9)	15.21 (13.64, 16.81)	54.2 (48.7, 60.3)	15.75 (14.16, 17.54)	−0.42 (−0.72, −0.11)
70+ years	13.7 (12.5, 15.1)	35.38 (32.31, 39.02)	39.2 (35.9, 42.6)	39.80 (36.47, 43.23)	0.30 (0.18, 0.43)
*Liver cancer due to alcohol use*					
Sex					
Male	14.2 (11.3, 17.6)	3.07 (2.48, 3.77)	35.2 (28.2, 43.9)	3.56 (2.90, 4.40)	0.03 (−0.20, 0.26)
Female	4.4 (3.4, 5.6)	0.98 (0.77, 1.23)	8.1 (6.5, 10.3)	0.80 (0.65, 1.01)	−1.22 (−1.42, −1.02)
Age^a^					
15‐49 years	4.5 (3.2, 6.3)	0.67 (0.47, 0.94)	6.4 (4.3, 9.4)	0.86 (0.58, 1.26)	−0.20 (−0.71, 0.31)
50‐69 years	9.8 (7.3, 12.8)	6.34 (4.77, 8.33)	24.3 (18.3, 32.4)	7.06 (5.32, 9.42)	−0.22 (−0.54, 0.10)
70+ years	4.3 (3.4, 5.4)	11.14 (8.90, 13.98)	12.7 (10.3, 15.9)	12.85 (10.43, 16.12)	0.26 (0.09, 0.42)
*Liver cancer due to NASH*					
Sex					
Male	7.7 (6.5, 8.8)	1.77 (1.53, 2.01)	21.6 (18.8, 24.5)	2.30 (2.01, 2.59)	0.48 (0.25, 0.71)
Female	4.8 (4.0, 5.6)	1.11 (0.93, 1.29)	10.1 (8.8, 11.6)	1.04 (0.91, 1.19)	−0.54 (−0.69, −0.39)
Age^a^					
15‐49 years	2.8 (2.3, 3.2)	0.41 (0.35, 0.48)	3.8 (3.2, 4.6)	0.51 (0.43, 0.62)	−0.49 (−1.08, 0.11)
50‐69 years	5.9 (5.0, 6.9)	3.83 (3.23, 4.50)	15.3 (12.8, 17.6)	4.45 (3.72, 5.12)	−0.04 (−0.36, 0.28)
70+ years	3.8 (3.2, 4.6)	9.93 (8.35, 11.81)	12.6 (10.7, 14.8)	12.83 (10.85, 15.00)	0.81 (0.67, 0.94)
*Liver cancer due to other causes*					
Sex					
Male	14.2 (12.0, 16.1)	2.86 (2.43, 3.23)	30.7 (26.9, 34.8)	3.15 (2.78, 3.55)	−0.21 (−0.45, 0.04)
Female	10.6 (9.0, 12.2)	2.28 (1.93, 2.62)	16.1 (14.3, 18.4)	1.63 (1.44, 1.86)	−1.66 (−1.84, −1.47)
Age^a^					
15‐49 years	9.0 (7.5, 10.6)	1.35 (1.12, 1.59)	10.7 (9.0, 12.6)	1.43 (1.21, 1.69)	−0.98 (−1.53, −0.42)
50‐69 years	11.0 (9.3, 12.7)	7.17 (6.05, 8.26)	24.2 (20.7, 28.2)	7.02 (6.01, 8.19)	−0.52 (−0.75, −0.28)
70+ years	4.4 (3.7, 5.2)	11.4 (9.63, 13.40)	11.9 (10.2, 13.8)	12.10 (10.36, 13.99)	0.13 (0.001, 0.25)

Abbreviations: ASIR: age‐standardized incidence rate; CI: confidence interval; EAPC: estimated annual percentage change; NASH: nonalcoholic steatohepatitis; UI: uncertainty interval.

a Crude mortality rate in each age group.

**Table 4:** The number of deaths and age‐standardized mortality rates of primary liver cancer by etiology in 1990 and 2017 and the temporal trends from 1990 to 2017

Characteristics	1990		2017		1990‐2017
	Deaths No. ×10^3^ (95% UI)	ASMR per 100 000 No. (95% UI)	Deaths No. ×10^3^ (95% UI)	ASMR per 100 000 No. (95% UI)	EAPC in ASMR No. (95% CI)
*Liver cancer due to hepatitis B*					
Sex					
Male	110.8 (99.5, 119.4)	22.70 (20.37, 24.46)	180.3 (167.2, 194.8)	18.16 (16.86, 19.60)	−1.17 (−1.39, −0.97)
Female	35.7 (30.9, 40.0)	7.63 (6.62, 8.54)	46.1 (42.1, 50.8)	4.62 (4.22, 5.09)	−2.07 (−2.24, −1.89)
Age^a^					
15‐49 years	51.8 (46.5, 55.9)	7.75 (6.96, 8.37)	46.8 (43.0, 50.4)	6.29 (5.78, 6.77)	−1.81 (−2.31, −1.30)
50‐69 years	69.9 (64.2, 74.9)	45.40 (41.64, 48.61)	124.4 (115.4, 134.1)	36.15 (33.55, 38.99)	−1.04 (−1.23, −0.85)
70+ years	24.0 (21.9, 26.2)	62.15 (56.68, 67.81)	55.0 (51.0, 59.4)	55.84 (51.75, 60.36)	−0.44 (−0.74, −0.14)
*Liver cancer due to hepatitis C*					
Sex					
Male	25.8 (22.9, 28.7)	6.38 (5.67, 7.04)	58.2 (53.1, 63.7)	6.33 (5.80, 6.91)	−0.37 (−0.58, −0.16)
Female	16.3 (21.3, 18.8)	4.53 (3.95, 5.13)	32.1 (29.3, 35.5)	3.31 (3.02, 3.66)	−1.27 (−1.40, −1.13)
Age^a^					
15‐49 years	5.6 (4.8, 6.3)	0.83 (0.72, 0.94)	5.9 (5.1, 6.7)	0.79 (0.69, 0.89)	−1.07 (−1.56, −0.58)
50‐69 years	23.0 (20.6, 25.4)	14.90 (13.35, 16.47)	43.9 (39.7, 48.5)	12.75 (11.53, 14.11)	−0.97 (−1.25, −0.69)
70+ years	16.1 (14.7, 17.8)	41.68 (38.11, 46.07)	40.6 (37.4, 44.0)	41.18 (37.96, 44.71)	−0.18 (−0.38, 0.02)
*Liver cancer due to alcohol use*					
Sex					
Male	13.8 (11.0, 17.1)	3.09 (2.51, 3.79)	28.3 (22.9, 35.3)	2.92 (2.39, 3.58)	−0.62 (−0.82, −0.43)
Female	4.5 (3.5, 5.7)	1.02 (0.81, 1.29)	7.5 (6.1, 9.4)	0.75 (0.61, 0.94)	−1.47 (−1.65, −1.28)
Age^a^					
15‐49 years	3.8 (2.6, 5.3)	0.56 (0.39, 0.79)	3.9 (2.7, 5.8)	0.53 (0.36, 0.78)	−1.17 (−1.63, −0.70)
50‐69 years	9.5 (7.2, 12.5)	6.19 (4.67, 8.10)	19.3 (14.7, 25.5)	5.61 (4.26, 7.43)	−0.83 (−1.11, −0.55)
70+ years	5.0 (4.0, 6.2)	12.87 (10.27, 16.12)	12.6 (10.3, 15.7)	12.80 (10.41, 15.97)	−0.29 (−0.53, −0.06)
*Liver cancer due to NASH*					
Sex					
Male	7.5 (6.4, 8.6)	1.82 (1.57, 2.08)	17.9 (15.7, 20.2)	1.96 (1.72, 2.20)	−0.15 (−0.37, 0.06)
Female	5.0 (4.2, 5.8)	1.19 (1.00, 1.39)	9.7 (8.5, 11.1)	1.01 (0.88, 1.16)	−0.76 (−0.91, −0.61)
Age^a^					
15‐49 years	2.2 (1.9, 2.6)	0.33 (0.28, 0.39)	2.3 (2.0, 2.8)	0.31 (0.26, 0.38)	−1.34 (−1.89, −0.80)
50‐69 years	5.8 (4.9, 6.8)	3.75 (3.16, 4.41)	12.3 (10.4, 14.2)	3.58 (3.02, 4.13)	−0.60 (−0.90, −0.31)
70+ years	4.5 (3.8, 5.4)	11.70 (9.87, 13.90)	13.0 (11.0, 15.2)	13.19 (11.19, 15.38)	0.31 (0.11, 0.51)
*Liver cancer due to other causes*					
Sex					
Male	13.0 (11.0, 14.8)	2.74 (2.33, 3.09)	23.5 (20.8, 26.6)	2.44 (2.16, 2.74)	−0.87 (−1.06, −0.68)
Female	10.3 (8.7, 11.9)	2.28 (1.94, 2.62)	14.4 (12.8, 16.5)	1.46 (1.30, 1.68)	−1.87 (−2.05, −1.69)
Age^a^					
15‐49 years	7.2 (5.9, 8.5)	1.08 (0.89, 1.28)	6.5 (5.5, 7.6)	0.87 (0.74, 1.02)	−1.82 (−2.33, −1.30)
50‐69 years	10.7 (9.1, 12.4)	6.96 (5.88, 8.05)	19.3 (16.6, 22.4)	5.60 (4.82, 6.52)	−1.10 (−1.32, −0.88)
70+ years	5.1 (4.4, 6.0)	13.32 (11.30, 15.64)	12.1 (10.5, 14.0)	12.33 (10.64, 14.21)	−0.37 (−0.56, −0.17)

Abbreviations: ASIR: age‐standardized incidence rate; CI: confidence interval; EAPC: estimated annual percentage change; NASH: nonalcoholic steatohepatitis; UI: uncertainty interval.

a Crude mortality rate in each age group.

### 3.3 Liver cancer due to hepatitis C

In 2017, 103.1 thousand new cases of liver cancer due to hepatitis C were reported, and these cases accounted for 20.0% of the total liver cancer cases (Table 1). The ASIR of liver cancer due to hepatitis C showed a small decrease from 1990 to 2017 (EAPC = −0.22, 95% CI −0.39, −0.04), and a corresponding decrease from 5.44 per 100 000 in 1990 to 4.77 per 100 000 in 2017 was found in the ASMR, with an EAPC of −0.79 (95% CI −1.37, −0.21) (Table 2). The proportion of liver cancer due to hepatitis C among males increased from 14.2% in 1990 to 17.6% in 2017 (Figure 3), and as expected, the ASIR increased from 6.09 per 100 000 to 7.38 per 100 000 during the same timeframe (EAPC = 0.30, 95% CI 0.10, 0.49) (Figure 1A, Table 3). However, the ASMR showed a significant downward trend among males (EAPC = −0.38, 95% CI −0.58, ‐ 0.16) (Figure 1B, Table 4). Among females, the ASIR and ASMR of liver cancer due to hepatitis C were reduced, with EAPCs of −1.02 (95% CI −1.15, −0.89) and −1.27 (95% CI −1.40, −1.13), respectively (Figure 1A,B). The incidence of liver cancer due to hepatitis C significantly increased in people aged ≥70 years and significantly decreased in people aged 50‐69 years, but the incidence of liver cancer due to hepatitis C remained stable among people aged 15‐49 years (Figure 2A, Table 3). A substantial reduction in mortality was found among people aged 15‐49 years and 50‐69 years but not among people aged ≥70 years (Figure 2B, Table 4).

### 3.4 Liver cancer due to alcohol use

In 2017, 43.3 thousand cases and 35.8 thousand deaths related to liver cancer due to alcohol use were reported, accounting for 8.4% and 8.6% of the total liver cancer cases and deaths, respectively. A minor decrease was found in the ASIR of liver cancer due to alcohol use from 1990 to 2017, with an EAPC of −0.27 (95% CI −0.50, −0.05) (Figure 1A, Table 1); however, the ASMR of liver cancer due to alcohol use remained stable during the same period (Figure 1B, Table 2). No significant trend in the ASIR of males was found, although a decrease was observed from 1990 to 2009 (EAPC = −0.25, 95% CI −0.48, −0.01), but a pronounced increase was observed from 2010 to 2017 (EAPC = 2.98, 95% CI 2.39, 3.57) (Figure 1A). In contrast, a significant downward trend was found in the ASMR (EAPC = −0.62, 95% CI −0.82, −0.43) (Figure 1B, Table 4). Among females, a significant decrease was observed in the ASIR from 1990 to 2009 (EAPC = −1.54, 95% CI −1.74, −1.35), which was followed by a marked increase from 2010 to 2017 (EAPC = 1.29, 95% CI 0.87, 1.71) (Figure 1A). A consistent downward trend in the ASMR was observed during the study period (EAPC = −1.47, 95% CI −1.65, −1.28) (Figure 1B, Table 4). The ASIR was significantly higher among people aged ≥70 years, while it remained stable in the other two age groups (Figure 2A, Table 3). The ASMR significantly decreased in all three age groups (Figure 2B, Table 4).

### 3.5 Liver cancer due to NASH

A total of 31.7 thousand cases and 27.6 thousand deaths related to liver cancer due to NASH were reported in 2017, accounting for 6.1% and 6.6% of the total liver cancer cases and deaths, respectively (Tables 1 and 2). Overall, the ASIR and ASMR of liver cancer due to NASH remained stable from 1990 to 2017 (Figure 3). The proportion of liver cancer due to NASH among males increased from 4.2% in 1990 to 5.5% in 2017, and a significant increase was found in the ASIR (EAPC = 0.48, 95% CI 0.25, 0.71) but not in the ASMR. The proportion of liver cancer due to NASH increased from 6.4% in 1990 to 8.4% in 2017 among females (Figure 3), and both the ASIR and ASMR decreased during the study period (Figure 1, Tables 3 and 4). Significant increases in the ASIR and ASMR were found in people aged ≥70 years (EAPC = 0.81 [95% CI 0.67, 0.94]; EAPC = 0.31 [95% CI 0.11, 0.51], respectively). However, the ASIR remained stable in other two age groups over time, and the ASMR decreased during the same timeframe (Tables 3 and 4, Figure 2A).

### 3.6 Liver cancer due to other causes

In 2017, 46.8 thousand cases and 37.9 thousand deaths were reported for liver cancer due to other causes, accounting for 9.1% and 9.1% of the total liver cancer cases and deaths, respectively (Tables 1 and 2). The ASIR and ASMR of liver cancer due to other causes decreased from 1990 to 2017, with EAPC values of −0.81 (95% CI −1.03, −0.59) and −1.35 (95% CI −1.76, −0.95), respectively. Among males, the proportion of liver cancer due to other causes remained relatively stable from 1990 to 2017 (Figure 3). Likewise, the ASIR remained steady during the same period, although the ASMR showed a significant decrease (Tables 3 and 4). Among females, the proportion of liver cancer due to other causes decreased slightly from 14.1% in 1990 to 13.3% in 2017, and the ASIR and ASMR decreased significantly by 1.66% and 1.87% per year, respectively, from 1990 to 2017 (Figure 3, Tables 3 and 4). A significant increase in the ASIR was found among people aged ≥70 years, while a significant decrease was observed among people aged 15‐49 and 50‐69 years (Figure 2A, Table 3). A marked reduction in the ASMR was found in all three age groups (Figure 2B, Table 4).

## 4 DISCUSSION

China is one of the countries most affected by liver cancer worldwide.^1^, ^20^ In this study, we comprehensively analyzed temporal trends in the incidence and mortality of liver cancer caused by distinct etiologies in China. In general, the incidence and mortality rates showed decreases over the last three decades, and the results were consistent in both sexes, most of the age groups, and most of the etiologies that we examined. The decrease was more substantial among females than among males. The most significant reduction was found among people aged 15**‐**49 years. Among the etiologies investigated in this study, liver cancer due to hepatitis B showed the most pronounced decrease.

The major risk factors for primary liver cancer that have been identified can be categorized into three groups^6^: (a) established factors, including infection with HBV or HCV, alcohol consumption, dietary aflatoxins, NAFLD, NASH,^25^ and smoking^26^; (b) likely factors, including diabetes mellitus, inherited metabolic disorders, hemochromatosis, porphyria cutanea tarda, and cirrhosis of any etiology; and (c) possible factors, such as low vegetable intake,^27^ oral contraceptive use,^28^ and radiation exposure.^29^ Chronic HBV infection in China has been considered to be the most common factor in the etiology of liver cancer and a major contributor to its development.^30^ Our study showed that the proportion of liver cancer caused by hepatitis B was approximately 60%, which is significantly lower than previous reports.^8^, ^31^ This difference might be partly explained by that we used the nationwide data instead of the regional data used in previous studies. To combat HBV, the Chinese government initiated a national immunization program to address the HBV epidemic. In 1999, the National Expanded Program on Immunization review reported that the immunization coverage with three doses of the HBV vaccine was 70.7%.^32^ Moreover, the “free‐of‐charge” and “catch‐up” HBV‐vaccination programs targeting all newborns and children aged 8‐15 years commenced in 2005 and 2009, respectively. All these efforts have significantly reduced the HBsAg carrier rate in China. In line with the decreased HBV infection rate, we found a decrease in the incidence of liver cancer due to hepatitis B, especially among middle‐aged people. However, no significant decrease was found in the incidence of liver cancer due to hepatitis B among people aged ≥70 years, which might be explained by the poor control of HBV infection in this population. In our previous study,^16^ we found that the most pronounced increase in the reported incidence of HBV was among older people (aged ≥65 years) of both sexes from 2004 to 2014. This increase might be partly attributed to the rise in healthcare availability in recent years. More importantly, the study suggested that a more efficient and tailored prevention strategy to control HBV infection is urgently needed for this population.

HCV infection is an emerging public health concern for China.^16^ In this study, the overall incidence of liver cancer due to hepatitis C decreased from 1990 to 2017. However, a significant increase was found among males and people aged ≥70 years, which indicates that the prevention of liver cancer due to hepatitis C should become a higher priority in the near future. HCV increases the risk of liver cancer by inducing fibrosis and, eventually, cirrhosis.^27^ As obesity and diabetes increase the risk of HCV‐related cirrhosis, the increase in the incidence of liver cancer due to hepatitis C is likely to be driven by both new HCV infections^16^ and increases in obesity and diabetes.^33^, ^34^ Currently, no effective vaccine for HCV is available worldwide,^35^, ^36^ which has greatly hampered the prevention of HCV infection and liver cancer due to hepatitis C. Although the treatment of HCV infection has progressed considerably as a result of the implementation of interferon‐free, direct‐acting antiviral (DAA)‐based combination therapies, the resistance of HCV to DAAs has played an important role in the failure of interferon‐free treatment regimens.^37^ Moreover, those fortunate enough to be cured of chronic hepatitis C with DAAs may remain at risk for liver cancer for an indefinite period.^38^, ^39^ Consequently, the prevention of liver cancer due to hepatitis C should involve at least three aspects: (a) preventing widespread transmission of HCV among the general population, (b) continuously investing in the development of the HCV vaccine, and (c) strengthening programs for weight management and increasing the rate of antiviral treatment for HCV as these drugs become less expensive to procure.

Alcohol use and NASH are widely known risk factors for the development of liver cancer.^17^, ^25^ According to a previous global survey,^40^ the adult per capita consumption of alcohol is moderate in China compared with that in European countries. Nevertheless, alcohol misuse among adolescents is a common issue worldwide and is an emerging problem in China.^41^ In this study, the incidence of liver cancer due to alcohol use showed a small decrease over the last three decades. Among males and people aged 15‐49 years, however, the downward trend has disappeared. The reduction in alcohol consumption observed since the 1970s in several countries across central Europe likely contributed to the decrease in liver cancer due to alcohol use incidence in that region.^42^ Therefore, policies intended to reduce alcohol consumption at the population level have the potential to substantially improve the health outcomes of populations, particularly young populations.^43^ NASH is a serious form of NAFLD, which can progress to advanced fibrosis, cirrhosis, and eventually hepatocellular carcinoma. In China, NAFLD is a relatively new public health concern, and the current prevalence of NASH among liver biopsy‐proven NAFLD patients is unknown.^44^ In this study, the incidence of liver cancer due to NASH was found to be stable during the study period. Future studies are needed to identify the risk factors for NASH in order to promote the prevention of liver cancer due to NASH.

Apart from the etiologies describe above, factors such as aflatoxin B1, tobacco smoking, and NAFLD were incorporated into the “other causes” category in this study. Aflatoxins are common contaminates of staple foods and have been found to be carcinogenic in many animal species, including humans.^45^ Aflatoxin exposure is clustered in high‐risk regions, where the combination of humid weather and improper food storage conditions expose the majority of the local population to aflatoxins, significantly increasing the incidence of liver cancer.^9^ The identification and removal of this risk factor has substantially decreased the incidence of liver cancer in young adults, suggesting the importance of controlling aflatoxin as a preventive measure.^46^ Tobacco smoking remains a severe public health concern in China, despite the implementation of tobacco control policies since the signing of the WHO Framework Convention on Tobacco Control in 2003.^47^ Therefore, action is needed to prevent further increases in smoking‐related chronic diseases, including liver cancer, as China's population ages. Notably, we found that the ASIR of liver cancer has risen in recent years. This result might be attributed to the increased availability of health care, which allows more patients to be identified. Furthermore, the aging population and the “lag‐effect” of HBV infection reservoir among older people might also contribute to the recent increase in liver cancer incidence.

The overall liver cancer mortality was found to be reduced in this study, which is consistent with the results of a previous study.^48^ The most pronounced decrease was found in liver cancer due to hepatitis B and in people aged 15‐49 years. As the health status of the aging population in China worsens, the burden caused by liver cancer mortality may continue to be a challenge for China in the future.

Our study has several limitations. First, multietiological liver cancer was not taken into account due to the scarcity of data in the GBD database. Second, the accuracy of the estimations might have been subject to the miscoding of liver metastases as primary liver cancer or the underreporting of liver cancer. Third, due to the limited access to the original data and data processing flow, we are dwarf in the evaluation of the data quality and validity. Moreover, all data in GBD study were estimated from mathematical models rather than observational data. Our results hence should be interpreted with cautions. Finally, in the current study, the data on liver cancer in China were estimated from the China DSP system and vital registration. Although the cancer registry system has covered about 25.27% of the national population by 2017,^49^ the improving data quality and changing population coverage should be taken into account when interpreting our results.

In summary, liver cancer remains a major public health concern in China. Since control of HBV infection improved, the incidence and mortality rates of liver cancer have significantly decreased over the past three decades. However, liver cancer due to hepatitis C, NASH, other causes, and other causes might be emergent concerns in the near future due to increases in the prevalence of their risk factors. The information provided by this study should help to illustrate the national landscape of liver cancer and establish additional targeted prevention strategies.

## CONFLICT OF INTEREST

We declare no conflicts of interest.

## AUTHOR CONTRIBUTIONS

Study design: TZ, XC, and LJ; Data collection: ZL, XM, and NC; Data analyses: ZL, XM, and YJ; Interpretation of the Results: All authors; Manuscript writing: ZL, TZ, XC, and XM; Manuscript proofreading: LJ, TZ, and XC.
